# Association Between Gut Microbiota and Osteoarthritis: A Review of Evidence for Potential Mechanisms and Therapeutics

**DOI:** 10.3389/fcimb.2022.812596

**Published:** 2022-03-16

**Authors:** Zhentian Wei, Feng Li, Guofu Pi

**Affiliations:** Department of Orthopedics, The First Affiliated Hospital of Zhengzhou University, Zhengzhou, China

**Keywords:** osteoarthritis, gut microbiota, immune system, metabolism, gut-brain axis

## Abstract

Osteoarthritis (OA) is a multifactorial joint disease characterized by degeneration of articular cartilage, which leads to joints pain, disability and reduced quality of life in patients with OA. Interpreting the potential mechanisms underlying OA pathogenesis is crucial to the development of new disease modifying treatments. Although multiple factors contribute to the initiation and progression of OA, gut microbiota has gradually been regarded as an important pathogenic factor in the development of OA. Gut microbiota can be regarded as a multifunctional “organ”, closely related to a series of immune, metabolic and neurological functions. This review summarized research evidences supporting the correlation between gut microbiota and OA, and interpreted the potential mechanisms underlying the correlation from four aspects: immune system, metabolism, gut-brain axis and gut microbiota modulation. Future research should focus on whether there are specific gut microbiota composition or even specific pathogens and the corresponding signaling pathways that contribute to the initiation and progression of OA, and validate the potential of targeting gut microbiota for the treatment of patients with OA.

## Introduction

Osteoarthritis (OA) is a multifactorial joint disease involving whole joint tissue dominated by articular cartilage damage, which eventually leads to pain, restricted movement of joints and even disability (Bijlsma et al., 2011). OA affects 303.1 million people globally and age-standardized prevalence, incidence, and years lived with disability for OA have increased by around 8-10% since 1990 (Safiri et al., 2020). The risk factors of OA include aging, gender, joint injury, diet, obesity, genetic predisposition and mechanical factors (Felson et al., 2000; Johnson and Hunter, 2014). The diagnosis and treatment recommendations of OA need the help from plain radiographs, diagnostic ultrasound and MRI (Abramoff and Caldera, 2020). Radiographic findings in OA include joint space narrowing, osteophytosis, subchondral sclerosis, and cyst formation (Abramoff and Caldera, 2020). The treatments for OA mainly include the modification of lifestyle, physical therapy, oral medications, injections and joint replacement surgery as well as some novel treatments such as mesenchymal stem cell injections, platelet-rich plasma injections and nerve blocks (Abramoff and Caldera, 2020). However, these treatments can only alleviate the symptoms of patients with OA. With the increase of obesity and aging population, the underlying trend of the incidence rate of OA is gradually upward, which causes an enormous economic burden on the world and severely affects the life quality of patients with OA. Therefore, it is essential to elucidate the etiology and pathogenesis of OA.

With the development of researches on OA, more and more researchers have found that the gut microbiota is closely related to OA. Gut microbiota is a collection of gut microbe populations, consisting of bacteria, fungi, viruses, phages, parasites and archaea, that colonizes the intestinal tract of the host and plays a key role in nutrient absorption, maintenance of metabolic homeostasis, development and maturation of immune system, resistance to infections, protection from development of systemic and mucosal immunity, and production of neurotransmitters (Kamada et al., 2013; Sommer and Bäckhed, 2013; Adak and Khan, 2019; de Sire et al., 2020). Gut microbiota, an “organ” that has endocrine and immune functions (Clarke et al., 2014; Lazar et al., 2018), is also a crucial component of the body ecosystem, which has a significant impact on people’s health.

Schott et al. (2018) showed that prebiotics reversed the effect of a high fat diet on OA by modulating gut microbiota, which suggested the impact of gut microbiota on OA. Guan et al. (2020) concluded that antibiotic induced gut microbiota dysbiosis could alleviate the progress of OA. The study of Ulici et al. (2018) showed that the severity of OA induced by destabilization of the medial meniscus was reduced in germ-free mice, compared to specific pathogen free mice, which suggested that the factors associated with the gut microbiota promoted the development of OA after joint injury. In fact, some clinical data have also confirmed the association between gut microbiota and OA. Huang et al. (2016) indicated that the levels of serum and synovial fluid lipopolysaccharide (LPS) derived from gut microbiota levels were associated with knee OA severity and inflammation. Boer et al. (2019) also found that the abundance of Streptococcus was associated with OA knee pain. Furthermore, Dunn et al. (2020) indicated that the content of gram- negative constituent bacterial DNA in both human OA cartilage and OA-susceptible mouse cartilage was increased, compared to human controls and OA-resistant mice, respectively. Interestingly, Zhao et al. (2018b) confirmed the presence of bacterial nucleic acids in synovial fluid and synovial tissue of patients with OA. Also, it is likely that these bacteria located in joints are translocated from gut microbiota *via* the damaged intestinal barrier. Given the evidences that gut microbiota is associated with OA, it is beneficial for the development of novel therapeutics for OA to clarify the mechanisms underlying the association. Consequently, this paper is going to review the potential mechanisms of the association between gut microbiota and OA from four aspects: immune system, metabolism, gut-brain axis and gut microbiota modulation. Also, it is hoped that this paper can help peers broaden the understanding of the pathogenesis of OA.

## Gut Microbiota Is Involved in the Development of OA Through the Immune System

OA has long been considered a degenerative disease of cartilage. In the past decade, however, our understanding of the underlying mechanisms of OA has changed fundamentally. We no longer regard OA as a typical degenerative disease caused by normal body wear, but a multifactorial disease, in which low-grade chronic inflammation plays a central role (Robinson et al., 2016). Now that it comes to inflammation, then the immune system is bound to be involved in the development of OA. At the same time, gut microbiota is also gradually considered as a potential driver of immune system activation (Dunn et al., 2020). It is promising that there has been accumulating evidence that the gut microbiota influences the progression of OA by affecting the body’s immune system or interacting with it.

### Gut Microbiota Affects the Intestinal Barrier

Intestinal barrier is the sum of the structure and function of the intestinal tract, which can prevent harmful substances such as bacteria and toxins from passing through the intestinal mucosa into other tissues, organs and blood circulation. The normal intestinal barrier consists of mechanical barrier, chemical barrier, immune barrier and biological barrier. Once the barrier is destroyed, it will lead to the leakage of intestinal contents that includes gut microbiota, its products and immune cells into the circulatory system, which probably contribute to endotoxin translocation and systemic inflammation. It has been shown that bacteria or related compounds (i.e., LPS and peptidoglycan) cross the intestinal barrier and enter the systemic circulation to mediate OA (Lorenzo et al., 2019). Based on the simultaneous assessment of the microbiome in the gut, blood and joints, Tsai et al. (2020) also made the hypothesis that a “leaky” gut allows microbiota, associated with dysregulation of immune gene signaling and promoting inflammation, to migrate from the gut to the joints, leading to the onset of OA. Therefore, the integrity of intestinal barrier is helpful to delay the progress of OA.

**Figure 1:**
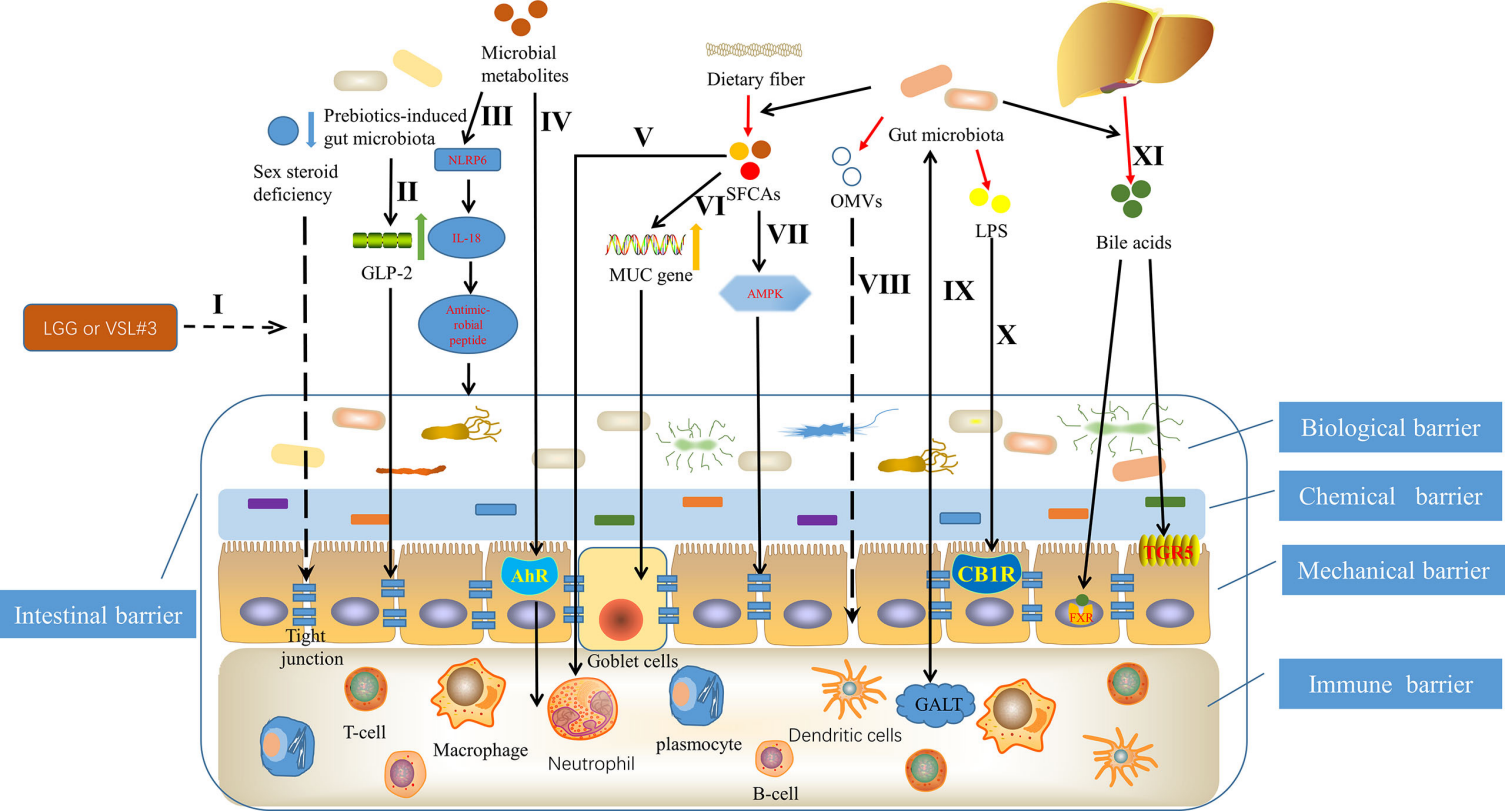
Mechanisms underlying that gut microbiota can affect the structure and function of intestinal barrier. I Probiotics Lactobacillus rhamnosus GG (LGG) or commercially available probiotic supplement VSL#3 enhanced the integrity of intestinal barrier through inhibiting the decreased expression of tight junction protein in intestinal epithelium, caused by sex steroid deficiency (Li et al., 2016a). II Prebiotics-induced gut microbiota improved intestinal barrier function, depending on glucagon-like peptide-2 (GLP-2) (Cani et al., 2009). III Microbial metabolites such as taurine, histamine and spermine, protected the intestinal barrier by shaping the host-microbiome interface through co-regulating NLRP6 inflammasome signaling, epithelial interleukin-18 (IL-18) secretion, and downstream antimicrobial peptide profiles (Levy et al., 2015). IVCommensal lactobacilli’s metabolites of tryptophan contributed to immune barrier by binding to aryl hydrocarbon receptor (AhR) (Zelante et al., 2013). V Short chain fatty acids (SCFAs), originating from fermentation of dietary fiber by gut microbiota (Ulici et al., 2018), promoted intestinal homeostasis through several hematopoietic cell types (Levy et al., 2017), such as neutrophil (Vinolo et al., 2011). VI SCFAs contributed to maintaining mucosal immunity by up regulating mucin (MUC) gene expression in intestinal epithelial goblet cells (Gaudier et al., 2004). VII SCFAs promoted tight junction assembly by activating adenosine monophosphate-activated protein kinase (AMPK) (Peng et al., 2009). VIII Outer membrane vesicles (OMVs), produced by pathogenic and commensal Gram-negative bacteria, disrupted the integrity of the mucosal epithelium (Kaparakis-Liaskos and Ferrero, 2015). IX The regulated cross talk between gut microbiota and gut-associated lymphoid tissue (GALT) contributed to the intestinal barrier (Kalinkovich and Livshits, 2019). X Lipopolysaccharide (LPS) regulated the intestinal barrier by activating intestinal cannabinoid type 1 receptor (CB1R) (Zoppi et al., 2012). XI Bile acids, an intestinal metabolite, protected the intestinal barrier, controlled by the gut microbiota through the farnesoid X receptor (FXR) and the G protein-coupled bile acid receptor 1 (GPBAR1) or (TGR5) (Levy et al., 2017).

More and more studies have proved that gut microbiota can affect the structure and function of intestinal barrier through a variety of mechanisms (**Figure 1**). Szychlinska et al. (2019) showed that dysbiosis, that is, the change of gut microbiota, can promote the excessive porosity of intestinal barrier if it lasts for a period of time. Li et al. (2016a) found that adding the commonly used probiotics *Lactobacillus rhamnosus* GG or commercially available probiotic supplement VSL#3 to the normal flora of sex steroid-deficient mice significantly enhanced the integrity of intestinal barrier and completely protect mice from bone loss caused by sex steroid deficiency. Strikingly, they also found that sex steroid deficiency resulted in decreased expression of tight junction protein in intestinal epithelium and increased permeability of epithelial barrier (Li et al., 2016a). Cani et al. (2009) found that changes in gut microbiota induced by prebiotics improved intestinal barrier function though a glucagon-like peptide-2 -dependent mechanism. Gut microbiota can induce the production of constitutive signaling, which can maintain the physiological inflammatory state of intestinal mucosa, continuously produce tissue repair factors, antibacterial proteins and immunoglobulin A (IgA), and jointly maintain the integrity of intestinal barrier (Rakoff-Nahoum et al., 2004; Sansonetti, 2004; Peterson et al., 2007). Kalinkovich and Livshits (2019) summarized that a vigorously regulated cross talk between gut microbiota, especially commensal bacteria and gut-associated lymphoid tissue, located in the intestinal lamina propria, promotes the integrity of intestinal barrier, ensures the function of intestinal epithelium and maintains intestinal immune homeostasis. Animal experimental data show that the activation of intestinal cannabinoid type 1 receptor *in vivo* can regulate the intestinal barrier function (Zoppi et al., 2012). Building on this, Liu et al. (2003) found that the gut microbiota, particularly through LPS and possibly nutrients, regulates the intestinal barrier by modulating the intestinal endocannabinoid system. Outer membrane vesicles (OMVs) are produced by pathogenic and commensal Gram-negative bacteria during their normal growth. The size of OMVs varies from about 20nm to 250nm. They are released from the bacterial membrane during the regulation of bacterial membrane proteins (Kulp and Kuehn, 2010). Kaparakis-Liaskos et al. (2015) indicated that based on the *in vitro* activity of OMVs, pathogens might use OMVs to disrupt the integrity of the mucosal epithelium, allowing bacterial components to enter the submucosa and interact directly with various immune cells, which include neutrophils, macrophages and dendritic cells, and in turn promote pathological changes.

Butyrate is a group of short chain fatty acids (SCFAs), which is produced in the lower intestine through fermentation of dietary fiber by gut microbiota (Ulici et al., 2018). Butyrate mediated by gut microbiota is the main energy source of colonic intestinal bacteria, which can reduce intestinal permeability and become a beneficial factor for intestinal health (Milani et al., 2017). In fact, Peng et al. (2009) have demonstrated that butyrate enhances intestinal barrier by activating adenosine monophosphate-activated protein kinase (AMPK) in Caco-2 cell monolayer to promote tight junction assembly. In addition, other studies have shown that SCFAs supplementation contributes to maintaining mucosal immunity through goblet cells, in which mucin gene expression is up regulated under butyrate (Gaudier et al., 2004). Likewise, SCFAs also promotes intestinal homeostasis through several hematopoietic cell types (Levy et al., 2017), which then helps to protect the structure and function of intestinal barrier. For example, neutrophil chemotaxis and leukocyte function are affected by SCFAs (Vinolo et al., 2011). There have been several studies suggesting the involvement of specific bacteria derived metabolites in the regulation of intraepithelial lymphocyte function (Lee et al., 2011; Li et al., 2011). Microbial metabolites of tryptophan represent a prime example (Lee et al., 2007; Li et al., 2014). Zelante et al. (2013) showed that by metabolizing tryptophan, commensal lactobacilli produced ligands for the aryl hydrocarbon receptor, which is a ligand activated transcription factor that is a powerful regulator of immune responses and has wide effects on the organogenesis, development of intestinal lymphoid follicle as well as the activation and proliferation of immune cells. Moreover, studies have found that the role of the aryl hydrocarbon receptor is essential in maintaining epithelial barrier and the homeostasis of intraepithelial lymphocytes (Lee et al., 2011; Li et al., 2011). More interestingly, Levy et al. (2015) demonstrated that taurine, histamine and spermine, the metabolites associated with the gut microbiota, shaped the host-microbiome interface by co-regulating NLRP6 inflammasome signaling, epithelial interleukin-18 (IL-18) secretion, and downstream antimicrobial peptide profiles, that is, helped to shape the intestinal microenvironment, of which the intestinal barrier is an important component. In addition, another intestinal metabolite, bile acids, produced by cholesterol in the liver and further metabolized by gut microbiota (de Aguiar Vallim et al., 2013), can regulate the growth of bacteria and protect the intestinal barrier, which is controlled by the gut microbiota through the farnesoid X receptor and the G protein-coupled bile acid receptor 1 (Levy et al., 2017). Taken together, a full appreciation of the mechanisms by which the gut microbiota affects the intestinal barrier may help to further deepen the link between the gut microbiota and OA, and in turn improve OA through precise interventions.

### Gut Microbiota Activates the Innate Immune System

Innate immunity refers to the host immune response induced by constant pattern recognition receptors (PRRs), which responds to conservative patterns in nature, including the immune response caused by invasive pathogens, such as bacteria, viruses and fungi (Kawai and Akira, 2010) It has been shown that the initiation and persistence of OA is closely related to the activation of the innate immune system (Scanzello et al., 2008). There’s evidence that changes in gut microbiota can activate the innate immune system, leading to increased production of pro-inflammatory cytokines, which could affect joints (Arend and Firestein, 2012; Huang and Kraus, 2016). It has also been confirmed recently that microbiota and microbiota-related molecules affect the pathogenesis of OA at both systemic and local levels through mechanisms involved in innate immune activation, in animal models (Liu et al., 2019).

**Figure 2:**
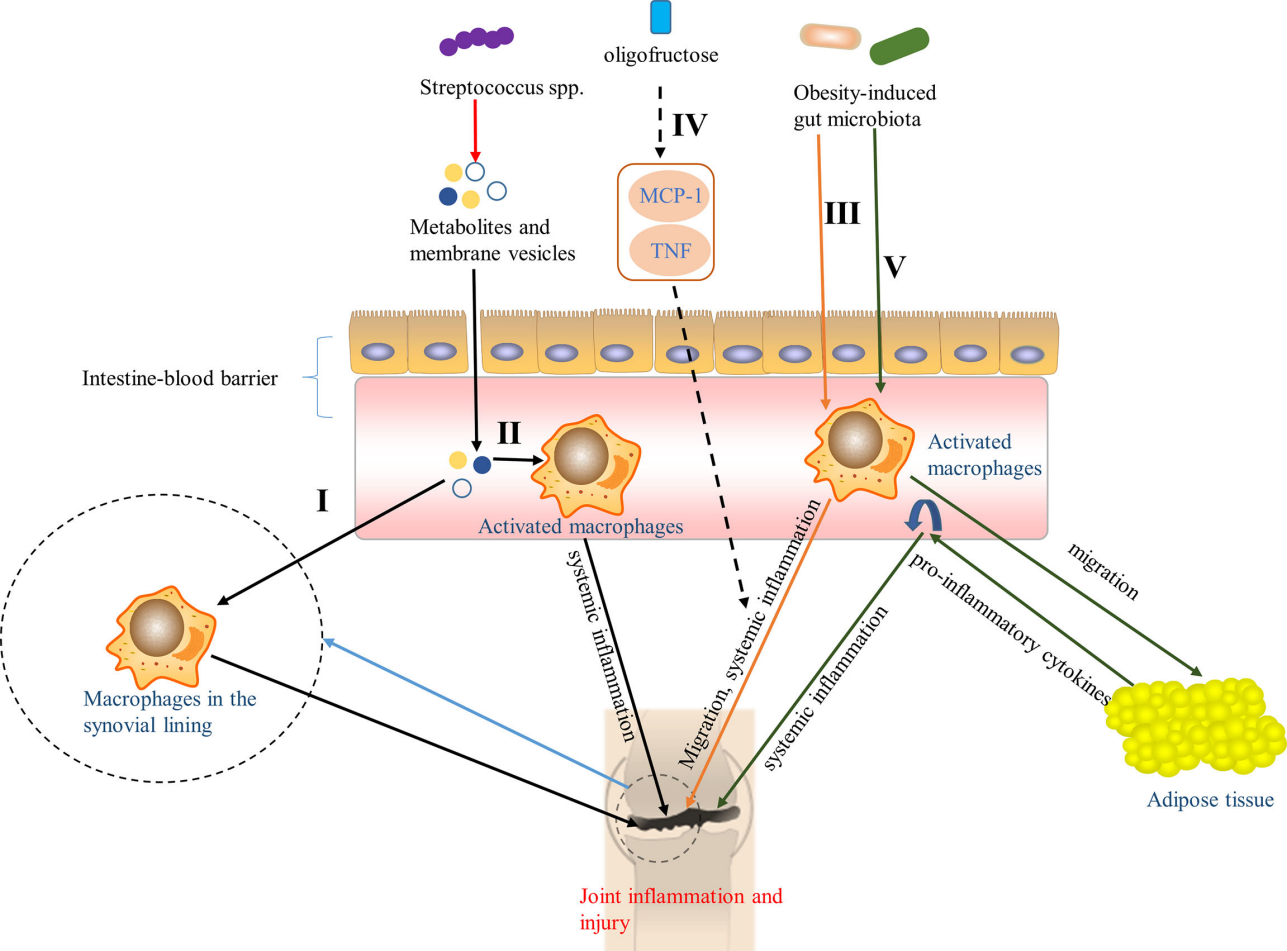
Pathways of the effect of gut microbiota on OA *via* macrophages. I Metabolites and membrane vesicles produced by Streptococcus spp. caused joint inflammation and injury by passing through the intestine-blood barrier to activate macrophages in the synovial lining (Boer et al., 2019). II Metabolites and membrane vesicles in the circulation, produced by Streptococcus spp., activated macrophages to pro-inflammatory macrophages and triggered a low-level systemic inflammatory state that aroused or aggravated joint inflammation and injury (Boer et al., 2019). III Obesity-induced gut microbiota accelerated knee OA by contributed to the systemic inflammation and the migration of macrophages to the synovium (Schott et al., 2018). IV Oligofructose, an indigestible prebiotic fiber, can reversed the pathway in III through inhibiting the up-regulation of monocyte chemotactic protein 1 (MCP-1) and tumor necrosis factor (TNF) (Schott et al., 2018). V Obesity-induced gut microbiota activated inflammation (John and Mullin, 2016; Portune et al., 2017), causing activated macrophages to migrate to adipose tissue that released pro-inflammatory cytokines into the circulation (Weisberg et al., 2003; Xu et al., 2003; Lumeng et al., 2007), aggravating systemic inflammation, and promoting the development of OA.

Macrophage is an important component of innate immune system and plays an important role in the generation and progression of OA. Daghestani et al. (2015) have shown that macrophage-associated inflammation is a driving factor for structural damage and progression of OA. Kraus et al. (2016) also showed that there were activated macrophages and macrophage-associated inflammation in the knee joints of patients with knee OA, which were closely related to the symptoms and imaging severity of knee OA. Moreover, it is worth noting that they also indicated that macrophages were etiological factors for OA pain at systemic joint sites (Kraus et al., 2016). Boer et al. (2019) found that gut microbiota was widely associated with OA-related knee pain and Streptococcus spp., and the higher the relative abundance of Streptococcus spp., the more severe the knee pain, which was driven by local inflammation in the joint. Notably, they also hypothesized that members of Streptococcus spp. produced metabolites and membrane vesicles, which can pass through the intestine-blood barrier and possibly activate macrophages in the synovial lining, resulting in joint inflammation and injury, or enter the circulation, activating macrophages to pro-inflammatory macrophages, thus triggering a low-level systemic inflammatory state, which arouses or aggravates joint inflammation and injury (**Figure 2** , I, II). In addition, Schott et al. (2018) reported that obesity can change the gut microbiota in obese mice-that is the increased abundance of the key pro-inflammatory species, leading to the climax of downstream systemic inflammation, accompanied by the migration of macrophages to the synovium, and then accelerating knee OA, which can be reversed by oligofructose, an indigestible prebiotic fiber, *via* inhibiting the upregulation of monocyte chemotactic protein 1 (MCP-1) and tumor necrosis factor (TNF), two cytokines that induce the joint inflammation (**Figure 2** , III, IV). Furthermore, in obesity, the changes of gut microbiota activate inflammation (John and Mullin, 2016; Portune et al., 2017), causing activated macrophages and other inflammatory cells to migrate to adipose tissue that releases TNF and other pro-inflammatory cytokines into the circulation (Weisberg et al., 2003; Xu et al., 2003; Lumeng et al., 2007), which aggravates systemic inflammation and promotes the development of OA (**Figure 2** , V).

The effect of gut microbiota on host inflammation mainly depends on the pattern recognition receptors, especially Toll-like receptors (TLRs) and nucleotide-binding oligomerization domain (NOD)-like receptors (NLRs) (McPhee and Schertzer, 2015; Nagpal et al., 2016). Microbial associated molecular patterns (MAMPs) can be recognized by the pattern recognition receptors, for which MAMPs are the most common ligands. MAMPs include factors such as LPS, peptidoglycan (PGN) (Clarke et al., 2010), flagellin and bacterial cell-free DNA, which can cross the intestinal barrier into the systemic circulation and elicit pro-inflammatory responses in resident immune cells, as well as be delivered to the joint where they stimulate innate immune receptors in bone, cartilage, and synovium to trigger pro-inflammatory responses (Huang et al., 2016; Hernandez, 2017).

**Figure 3:**
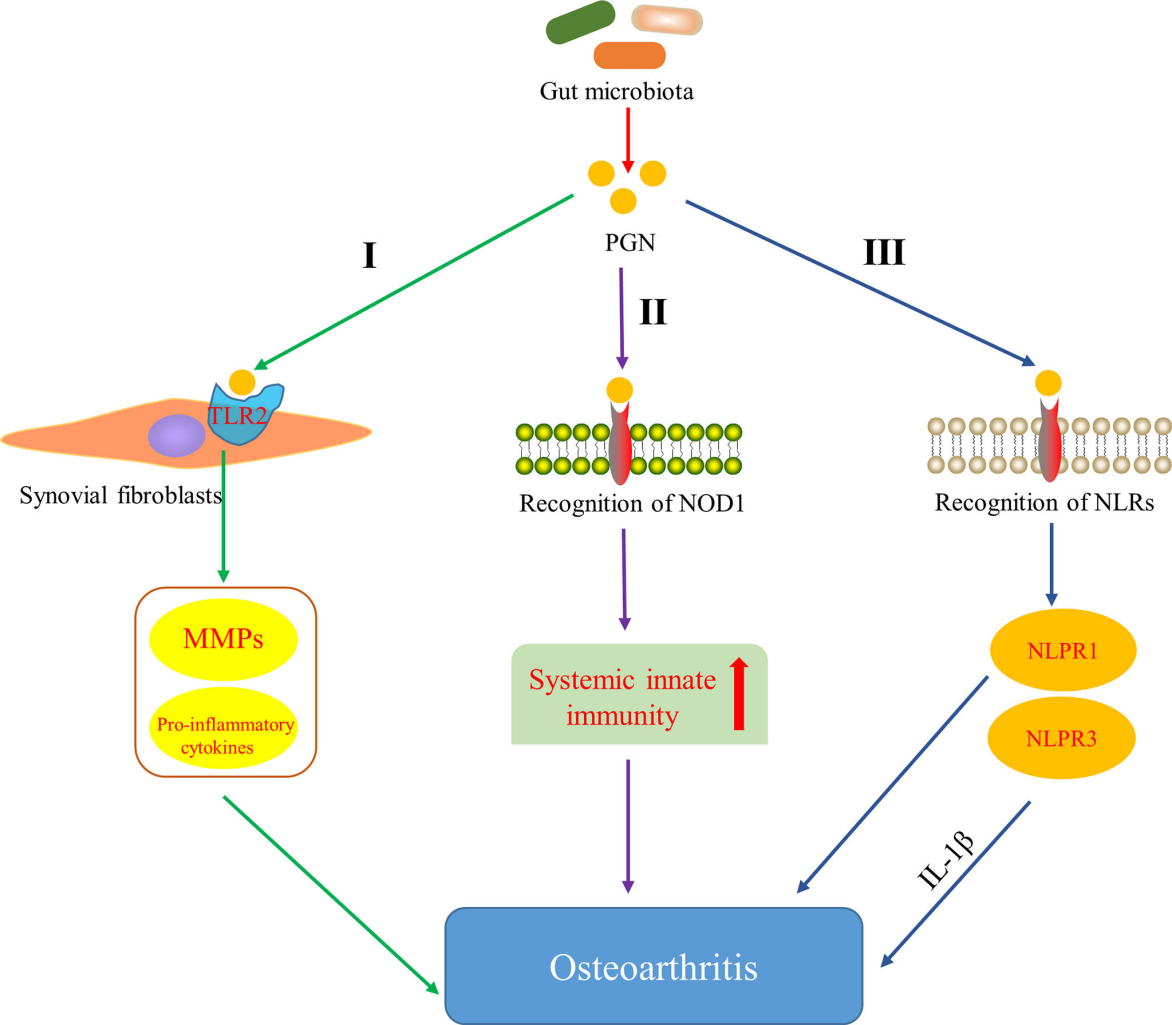
Pathways of peptidoglycan (PGN) involved in OA. I PGN affects the development of OA by inducing the expression of matrix metalloproteinases (MMPs) and pro-inflammatory cytokines through activating Toll-like receptor 2 (TLR2) on synovial fibroblasts (Kyburz et al., 2003). II PGN affects the development of OA by recognizing NOD-like receptor 1 (NOD1) and promoting systemic innate immunity (Clarke et al., 2010). III PGN affects the development of OA by recognizing NOD-like receptors (NLRs), activating NLRP1 and NLRP3 inflammasome, and promoting the increase of pro-inflammatory cytokines.

Several studies have indicated that bacterial PGN can stimulate intra-articular synovial fibroblasts and induce the expression of matrix metalloproteinases (MMPs) and pro-inflammatory cytokines by activating the outer membrane protein TLR2 on synovial fibroblasts (Kyburz et al., 2003) (**Figure 3** , I). Moreover, peptidoglycan-polysaccharide (PGN-PS) complexes have been detected in synovial underlay cells of OA-affected joints (van der Heijden et al., 2000) and confirmed the arthrogenic properties in adjuvant-induced arthritis model (Kool et al., 1991). Clarke et al. (2010) showed that gut microbiota-derived PGN initiated and enhanced systemic innate immunity through recognition of NOD1 (**Figure 3** , II). NLRs proteins are the essential components (or precursors) of inflammasome (Horng and Hotamisligil, 2011; Zhao et al., 2018a), which is related to the inflammatory pathway of the body. There are increasing evidences that NLRP3 inflammasome is a fresh biomarker for the diagnosis and monitoring of OA (McAllister et al., 2018) and Martinon et al. (2006) demonstrated that NLRP3 inflammasome in macrophages was engaged by calcium pyrophosphate dihydrate crystals to induce the release of pro-inflammatory cytokines interleukin-1-beta (IL-1β), which has been proved to inhibit production of cartilage extracellular matrix components, including types II and IX collagen (Goldring et al., 1988) and aggrecan (Pfander et al., 2004). In addition, the expression of NLRP1 inflammasome has also been found to be upregulated in the synovia of patients with OA (Zhao et al., 2018a). These data strongly indicate that inflammasome plays an important role in the pathology and progression of OA. It is well known that PGN derived from the gut microbiota can be used as the ligands of NLRs. Therefore, it is hypothesized that PGN is involved in the progression of OA through the pathway that PGN recognizes NLRs, activates the inflammasome and promotes the increase of pro-inflammatory cytokines (**Figure 3** , III). These results and hypotheses indicate that PGN is implicated in triggering and exacerbating OA.

**Figure 4:**
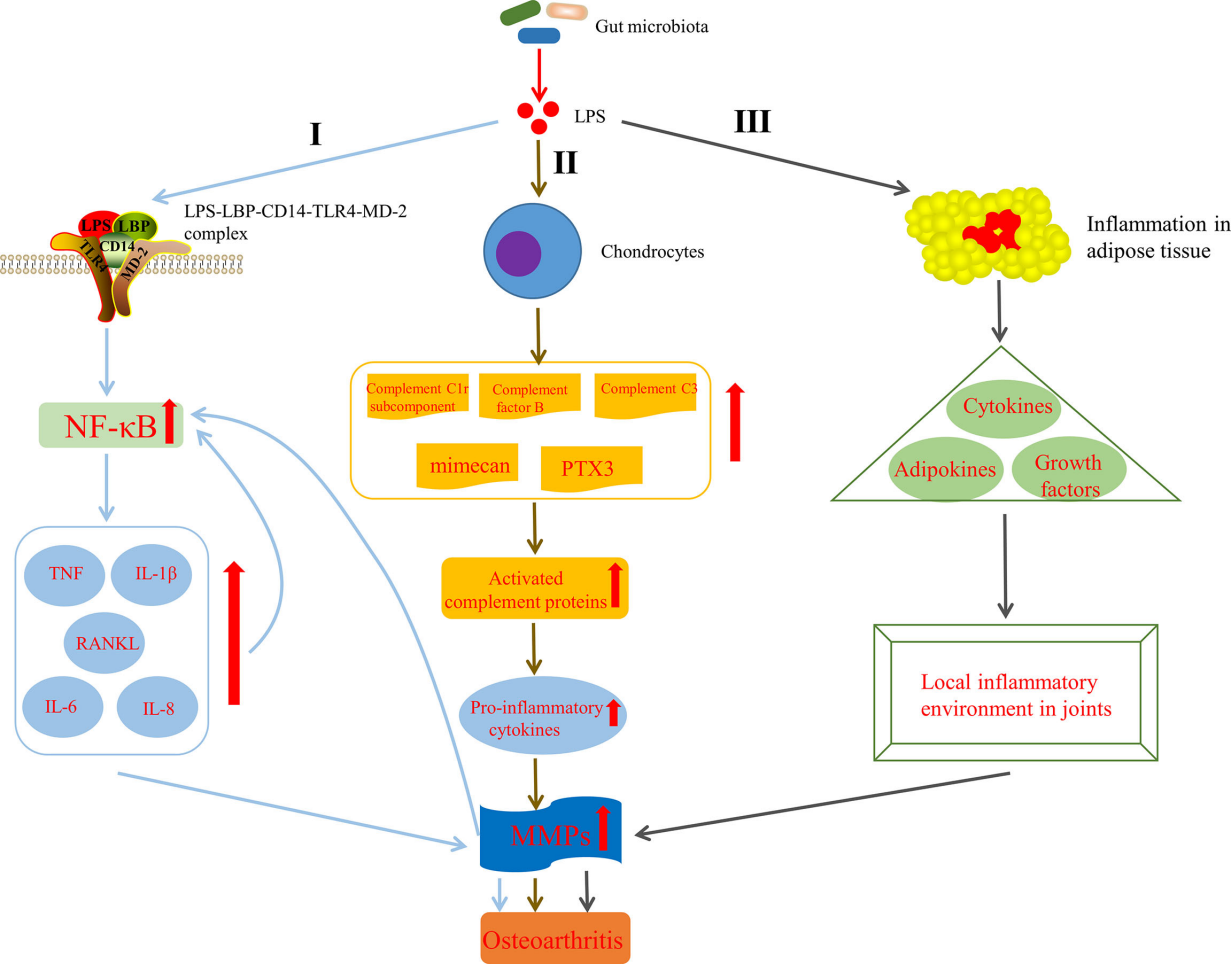
Mechanisms by which lipopolysaccharide (LPS) affects the development of OA. I LPS is involved in OA by activating innate immune response *via* the formation of the LPS–LBP–CD14–TLR4–MD-2 complex, increasing NF-κB levels, up regulating TNF, IL-1β, IL-6, IL-8 and RANKL levels, raising the production of MMPs and further reinforcing NF-κB activation. II LPS is involved in OA by activating complement pathway in chondrocytes (Haglund et al., 2008). III LPS is involved in OA by inducing inflammation in adipose tissue, precipitated systemic changes in cytokines, adipokines and growth factors, and finally affecting the local inflammatory environment inside the knee joint (Bleau et al., 2015). LPS, lipopolysaccharide; LBP, LPS binding protein; MD-2, myeloid differentiation protein-2; NF-κB, nuclear factor kappa-light-chain-enhancer of activated B cells; TNF, tumor necrosis factor; IL-1β, interleukin-1-beta; RANKL, receptor activator of NF-κB ligand; PTX3, long pentraxin 3; MMPs, matrix metalloproteinases.

Huang et al. (Huang and Kraus, 2016; Huang et al., 2016) indicated that the level of LPS (also known as endotoxin) was closely associated with the severity and inflammation of knee OA and formulated a two-hit model of OA pathogenesis, in where the first hit is that joint tissue macrophages are primed by LPS through TLR4 and the second one is that macrophage inflammasome pathways are activated by damage associated molecular patterns, resulted from structural joint damage. The model activates innate immunity and then promotes systemic and joint inflammatory response and joint structural damage *via* coexisting complementary mechanisms, such as inflammasome activation or assembly of fragmented cartilage matrix molecules (Huang and Kraus, 2016). It has been shown that LPS can activate macrophages and neutrophils in the innate immune system and induce them to synthesize pro-inflammatory factors, such as IL-1β and TNF, MMPs and free radicals, which lead to significant secondary inflammation in the joint tissue (Lorenz et al., 2013). Strikingly, one pathway through which LPS participate in the progression of OA has been discovered. CD14, existing in various cell types, mainly monocytes and macrophages (Landmann et al., 2000), acts as a receptor for LPS-LPS binding protein (LBP) complex (Wright et al., 1990), which plays an important role in the pathogenesis of OA. LPS activates innate immune response though the bind of the CD14–LPS–LBP complex and TLR4, expressed on the cell surface of various cell types, especially monocytes and other immune cells, as well as its co-receptor myeloid differentiation protein-2 (MD-2) (Akashi et al., 2000; Cani et al., 2007). This activation leads to the increased levels of nuclear factor kappa-light-chain-enhancer of activated B cells (NF-κB) (Nair et al., 2012). It is known that the increased NF-κB levels can induce activated joint cells to produce catabolic cytokines and chemokines, such as TNF, IL-1β, IL-6, receptor activator of NF-κB ligand (RANKL) and IL-8, which in turn can raise the production of MMPs, reduce the synthesis of collagen and proteoglycan, further reinforce NF-κB activation and finally end up in the secondary inflammation of the articular tissues (Kapoor et al., 2011) (**Figure 4** , I). What can be added is that some studies have shown that LPS can also induce the secretion of MMPs and components of the innate immune response in cultured chondrocytes (Haglund et al., 2008) and expedite the matrix degradation of articular cartilage explants (MacDonald et al., 1994). In addition, LPS has been demonstrated to activate chondrocytes to induce the production of complement C1r subcomponent, complement factor B, complement C3, mimecan (osteoglycin) and long pentraxin 3, leading to activation of the complement cascade and production of active complement proteins (Haglund et al., 2008), which can bind to receptors or deposit on synovial cells, leading to increased pro-inflammatory cytokines production(**Figure 4** , II). This pathway indicates that LPS not only induces innate immune response through TLR4 activation, but also aggravates the existing chronic low-grade inflammation by upregulating pro-inflammatory cytokines (Huang and Kraus, 2016), conferring OA to a chronic disease state (Scanzello et al., 2008). Interestingly, there is evidence that the intrinsic inflammatory mediators secreted by body fat or adipose tissue, including cytokines and adipokines, may lead to initiation and progression of OA (Kapoor et al., 2011; Berenbaum, 2013). Likewise, several *in vitro* studies have demonstrate that adipokines, including leptin, adiponectin, visfatin and resistin have an ability to induce inflammatory mediators and chondrolysis (Conde et al., 2011). On the basis of the above, Bleau et al. (2015) also indicated that LPS induced inflammation in adipose tissue and precipitated systemic changes in cytokines, adipokines, and growth factors, including leptin, IP-10 and IL-1α, which ultimately facilitated the development of OA by affecting the local inflammatory environment inside the knee joint (**Figure 4** , III). Moreover, it has been shown that LPS can interact with the TLR4–CD14 complex to stimulate signaling cascades and in turn induce the expression of TLR2, which further augments the innate immune response (Ojaniemi et al., 2006). Also note that LPS induced inflammation and pyroptosis in fibroblast-like synoviocytes derived from patients with OA, mediated by NLRP1 and NLRP3 inflammasomes (Zhao et al., 2018a). To sum up, LPS plays a pathogenic role in the perpetuated development of OA by activating the innate immune system and mediating the expression of various cytokines *via* multiple pathways.

### Gut Microbiota Influences the Adaptive Immunity

Previously, it seemed that OA had nothing to do with the adaptive immunity. Even if it has been discovered that the synovial tissue of OA includes some immune cells, such as B cells, plasma cells, mast cells and natural killer (NK) cells (de Lange-Brokaar et al., 2012; de Lange-Brokaar et al., 2016; Klein-Wieringa et al., 2016), but there is still a lack of researches to confirm the possible pathological role of these immune cells in the development of OA. However, recent evidences suggest that T cells play a role in the pathogenesis of OA. According to the researches of Qi et al. (2016) and Shan et al. (2017), it can be suggested that T cells are dysregulated in OA. Sadtler et al. (2016) found that helper T (Th) cells could induce macrophages to produce pro-regenerative phenotypes in an IL-4 dependent manner through the release of cytokines, which indicates that Th cells have a biological role in controlling inflammation and repair. Significantly, Li et al. (2017) have also reviewed the effect of T cells in OA, especially Th cells, such as Th1, Th2, Th9, Th17, follicular helper T (Tfh) Cells, regulatory T (Treg) cells and so on: T cells are clearly present in OA organs and produce catabolic cytokines that stimulate protease to destroy cartilage matrix or modulate the secretion of anti-inflammatory cytokines and the expression of receptors for cytokines. In addition, Th cells have potential to affect the progression of OA through regulating the adaptive immune system (Woodell-May and Sommerfeld, 2020).

**Figure 5:**
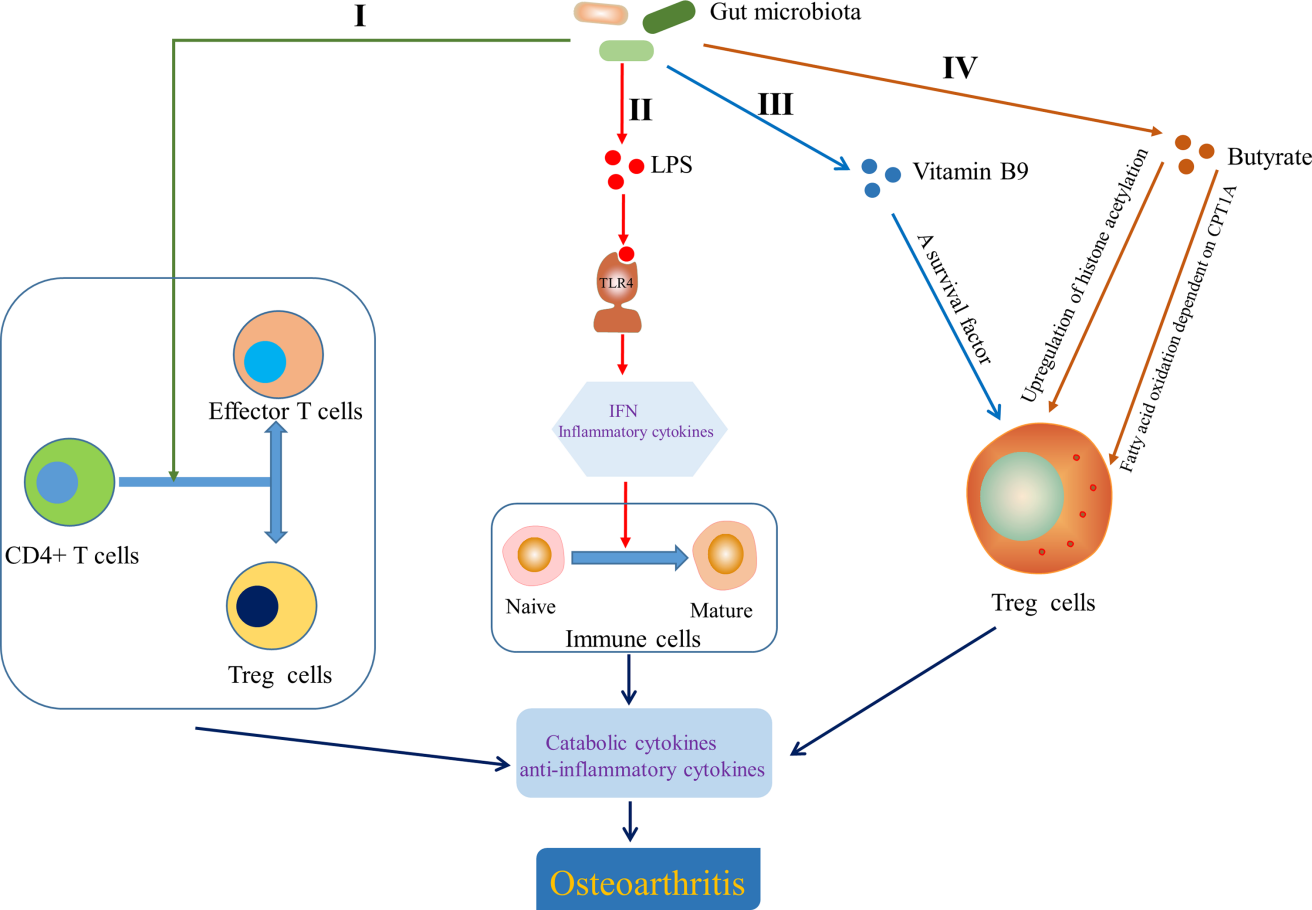
Effect of gut microbiota on the adaptive immunity. I Gut microbiota determined the direction of differentiation of primitive CD4+ T cells into effector T cells or Treg cells (Honda and Littman, 2012). II Lipopolysaccharide (LPS) helped to induce naive immune cells to mature immune cells by activating Toll-like receptor 4 (TLR4) to cause an inflammatory cascade (Chen et al., 2011; Chu and Mazmanian, 2013; Kim, 2013). III Vitamin B9 contributed to the survival of Treg cells (Kunisawa et al., 2012). IV Butyrate modulated the generation and differentiation of Treg cells by up regulating histone acetylation (Arpaia et al., 2015) and enhancing fatty acid oxidation dependent on carnitine palmitoyl transferase 1A (CPT1A) (Hao et al., 2021). IFN, interferon.

In view of the role of T cells in OA, attention has been paid to that gut microbiota is involved in the development of OA by influencing adaptive immunity. Gut microbiota dysbiosis can determine the direction of differentiation of primitive CD4+ T cells into effector T cells or Treg cells. The balance between Treg cells and effector T cells subsets Th1, Th2 and Th17 is crucial for immune homeostasis, the imbalance of which can lead to chronic inflammation, including joints (Honda and Littman, 2012) (**Figure 5** , I). Furthermore, although the role of Tfh cells in the pathogenesis of OA has not been clarified, Shan et al. (Shan et al., 2017) found that the percentages of CXCR5+CD4+ Tfh cells in OA patients were significantly higher than that in the healthy control group. Interestingly, a novel insight showed that the activation of CXCR5+CD4+ Tfh cells could be induced by gut microbiota and bile acid metabolism (Cheng et al., 2022). Segmented filamentous bacteria, a gut-residing specie, has been demonstrated to drive autoimmune arthritis *via* Th17 cells (Wu et al., 2010), which can be accumulated in the synovial fluid and synovial tissue of OA patients (Chabaud et al., 1999; Qi et al., 2016). So et al. demonstrated that Lactobacillus casei, a probiotic, suppressed rheumatoid arthritis (RA) by down-regulating Th1 type inflammatory responses (So et al., 2008) and the mixture of Lactobacillus casei with type II collagen/glucosamine inhibited the inflammatory activity of synovial fibroblasts or chondrocytes in experimental OA by regulating T cells response (So et al., 2011). LPS, as a MAMP, has been shown to activate TLR4 to cause an inflammatory cascade that releases interferons and inflammatory cytokines, which act as transcription factors to induce naive immune cells to mature (Chen et al., 2011; Chu and Mazmanian, 2013; Kim, 2013) (**Figure 5** , II). Interestingly, gut microbiota derived ligands differentially skewed the balance of T cells in a mouse model of arthritis that affects joint inflammation and cartilage and bone destruction by stimulating TLR2 and TLR4 (Abdollahi-Roodsaz et al., 2008). Vitamin B and K dependent on the microbiota for biosynthesis have been reported to influence the biology of T cells. For example, vitamin B9, the synthesis of which depend on Bifidobacterium and Lactobacillus, is a survival factor for Treg cells (Kunisawa et al., 2012) (**Figure 5** , III). Butyrate derived from gut microbiota is conducive to shaping the intestinal immune homeostasis by inducing the differentiation of colonic inducible Treg cells (Furusawa et al., 2013) through enhancing fatty acid oxidation dependent on carnitine palmitoyl transferase 1A (CPT1A) (Hao et al., 2021). Butyrate also promotes the generation of Treg cells by upregulating gene expression of histone acetylation (Arpaia et al., 2015) (**Figure 5** , IV). Moreover, Hui et al. (Hui et al., 2019) have demonstrated that butyrate inhibits collagen-induced arthritis *via* Treg cells/IL-10/Th17 cells axis, which also leads us to think whether butyrate could affect OA progression in the same pathway. Collectively, these data indicate that gut microbiota and related products also affect OA progression by influencing the adaptive immunity, particularly the regulation of T cells.

## Gut Microbiota Is Involved in the Development of OA Through the Metabolism

As we all know, the risk factors of OA include aging, diet and obesity, which are related to the metabolism of the body. Mooney et al. (2011) suggested that metabolic dysregulation is a comorbid factor in OA-related cartilage degeneration in a type 2 diabetic mouse model, induced by high-fat diet. Mobasheri et al. (2017) also indicated that OA acted as a metabolic disorder and metabolism play an important role in cartilage and synovial joint function, and they reviewed the effect of metabolism in the pathogenesis of OA. Moreover, Metabolic syndrome-associated OA (MetS-OA) is a significant clinical phenotype, which links metabolic diseases (obesity, diabetes and insulin resistance, dyslipidemia, and hypertension) to OA (Courties et al., 2017). Insulin resistance has also been found to be independently associated with OA (Silveri et al., 1994) and the association could be mediated through the secretion and maintenance of the cartilagenous matrix impaired by insulin resistance, and intracellular signalling cascades in chondrocytes induced by advanced glycation end-products(AGEs), a consequence of Type 2 diabetes mellitus (DeGroot et al., 2004). As a result, metabolism is closely related to OA. Interestingly, gut microbiota can be regarded as an independent endocrine organ, which is involved in maintaining host energy homeostasis and stimulating host immunity through a molecular crosstalk with the host (Clarke et al., 2014), and indirectly be implicated in the pathogenesis of obesity and metabolic diseases by triggering insulin resistance, low-grade inflammation and excess lipid accumulation in the host through molecular interactions with energy metabolism and inflammation pathways of the host (Boulange et al., 2016). Based on the above evidences, gut microbiota may be involved in the development of OA by affecting or interacting with the metabolism of the body.

### Energy Metabolism

**Figure 6:**
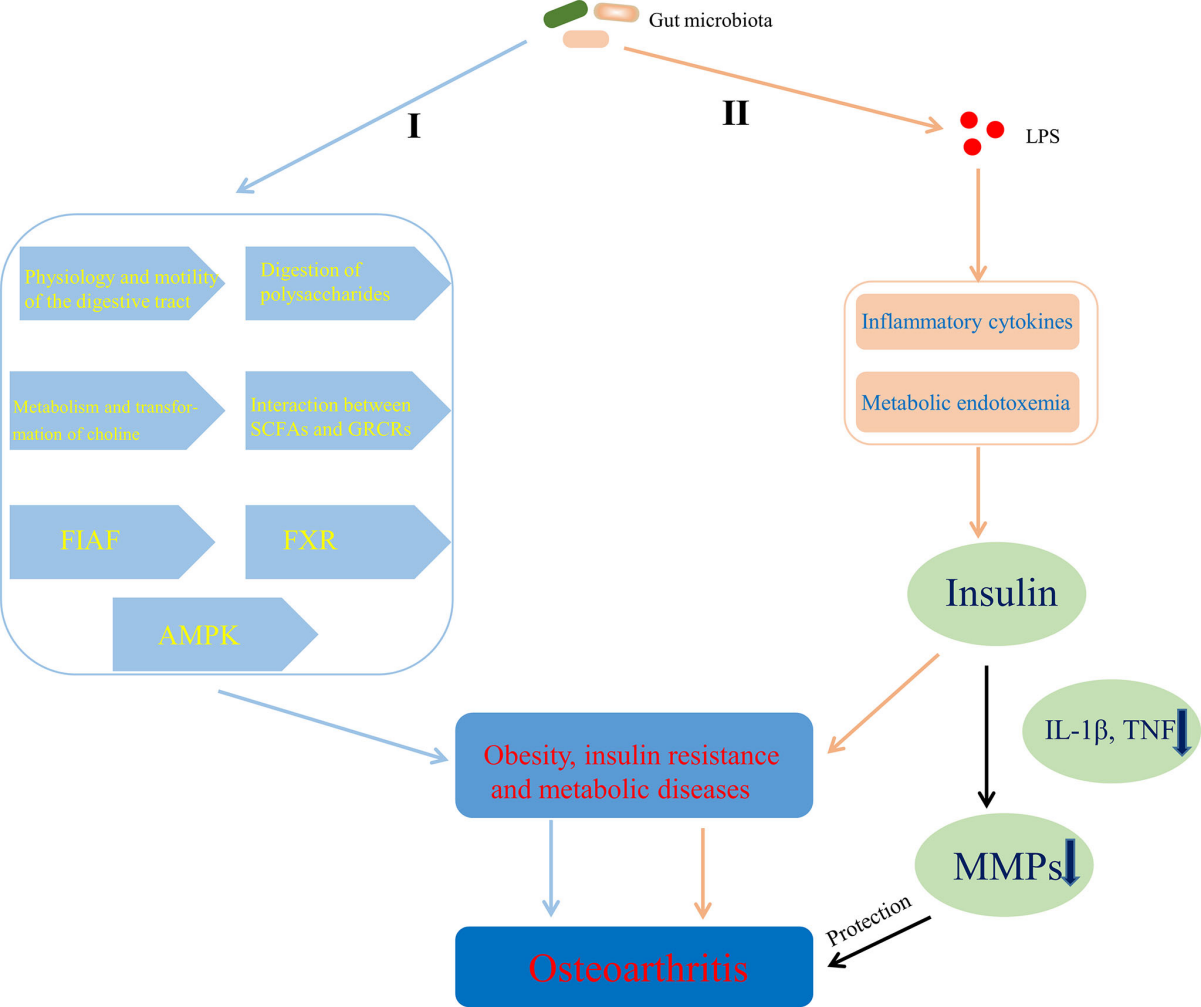
Mechanisms of gut microbiota’s contributing to OA through affecting energy metabolism. I Gut microbiota contributed to obesity and metabolic diseases by helping to obtain energy and increase host fat storage *via* pathways involved in the physiology and motility of the digestive tract, the digestion of polysaccharides, the metabolism and transformation of choline, the interaction between SCFAs and GPCRs, FIAF, FXR, and AMPK. II LPS-mediated inflammatory pathway or metabolic endotoxemia contributed to obesity and insulin resistance by affecting the regulation of insulin secretion that can protect articular cartilage through inhibiting MMPs expression dependent on pro-inflammatory cytokines. SCFAs, short chain fatty acids; GPCRs, G protein-coupled receptors; FIAF, fasting-induced adipose factor; FXR, farnesoid X receptor; AMPK, adenosine monophosphate-activated protein kinase; LPS, lipopolysaccharide; TNF, tumor necrosis factor; MMPs, matrix metalloproteinases.

There are some evidences that gut microbiota can help to obtain energy and increase host fat storage though participating in the physiology and motility of the digestive tract (Abrams and Bishop, 1967; Musso et al., 2011) and the digestion of polysaccharides (Gibson et al., 2004), inhibiting fasting-induced adipose factor (Bäckhed et al., 2004) in the intestine and AMPK (Winder and Hardie, 1999; Samuel et al., 2008), affecting the metabolism and transformation of choline (Noga and Vance, 2003; Dumas et al., 2006; Cole et al., 2012), regulating the farnesoid X receptor (Parséus et al., 2017) and modulating the interaction between SCFAs and G protein-coupled receptors (GPCRs; GPR41, GPR43, and GPR109A) (Bäckhed et al., 2004; Samuel et al., 2008; Bellahcene et al., 2013; Neves et al., 2015), which contributes to obesity and metabolic diseases (**Figure 6** , I). Chronic inflammation also linked gut microbiota to obesity and insulin resistance. Cani et al. (2007) suggested that LPS initiated the inflammation-related processes, which are associated with the onset of obesity and insulin resistance. Some studies also showed that LPS-mediated inflammatory pathway or metabolic endotoxemia played an important role in the physiology and pathology of obesity and insulin resistance by being involved in the regulation of insulin secretion (Shi et al., 2006; Ghanim et al., 2009; Vergès et al., 2014) that can protect articular cartilage by inhibiting pro-inflammatory cytokines (such as IL-1β, TNF)-dependent expression of cartilage-degrading enzymes, MMPs (Hamada et al., 2016) (**Figure 6** , II). Moreover, Adipose tissue, including one surrounding the articulation, is no longer considered a passive energy storage site but is a bona fide endocrine “organ” with the capacity to secrete adipokines such as leptin, resistin, visfatin and adiponectin (Zeddou, 2019), the ability of which to contribute to OA has been summarized by Metcalfe et al. (2012). Indeed, the level of leptin in the synovial fluid has been shown to be related to OA (Ku et al., 2009; Xiong et al., 2019a) and the leptin gene and its receptor gene have also been demonstrated to be associated with OA by single-nucleotide polymorphism analysis (Qin et al., 2010; Ma et al., 2013). It has also been noted that increased levels of leptin, binding to the leptin receptor (Ob-Rb), promote the expression of IL-6 through the Janus kinase 2/signal transducer and activator of transcription (JAK2/STAT3), p38 MAPK, or phosphatidylinositol 3-kinase/protein kinase B (PI3K/AKT) signaling pathways (Xiong et al., 2019b). Furthermore, Leptin could also enhance the expression of other factors, such as IL-1, MMP9, and MMP13 (Simopoulou et al., 2007; Troeberg and Nagase, 2012). Overall, the above evidences suggest that adipose tissue-derived adipokines, especially leptin (Yang et al., 2013), could induce the release pro-inflammatory cytokines and promote the progression of OA through cartilage damage. Interestingly, LPS derived from gut microbiota induces the increased expression of adipokines by stimulating adipose tissue (Grunfeld et al., 1996; Bleau et al., 2015), which is an important pathway for gut microbiota to be involved in the development of OA by affecting metabolism.

### Diet-Associated Factors Metabolism

Free fatty acids (FFAs) have been shown to play an important role in OA pathophysiology. Wu et al. (2017) indicated that serum and synovial fluid lipidomic profiles that are expected to predict obesity-associated OA could act as sensitive biomarkers of the radiographic stage of obesity-associated OA. Some studies suggested that metabolites related to the metabolism of FFAs in synovial fluid, such as myristic acid, oleic acid and lanosterol, were positively correlated with the structural deterioration of OA (Kim et al., 2017) and total fatty acids, especially arachidonic acid, were closely related to the severity of cartilage surface erosion (Lippiello et al., 1991). It was also observed that ectopic lipids were accumulated in the articular cartilage of patients with OA (Lippiello et al., 1991) and lipotoxic effects caused by dyslipidemia of major cells within synovial tissue, including macrophages and adipocytes, exacerbated synovitis in patients with OA and metabolic syndrome (Larrañaga-Vera et al., 2017). Furthermore, Lee et al. (2018) and his colleagues also showed that pathological concentrations of oleic acid could decrease the viability of articular chondrocytes *via* apoptosis and FFAs-induced lipotoxic effects in chondrocytes correlated with the amount of cellular FFAs that were initially sequestered in lipid droplets. It is noteworthy that imbalanced gut microbiota has been demonstrated to be related to the significantly increased expression of key genes related to FFAs synthesis and FFAs transport in liver (Jin et al., 2016) and specific gut microbiota signatures, particularly imbalanced populations of Akkermansia and Lactobacillus, have been discovered to be associated with altered serum FFAs profiles (Rodríguez-Carrio et al., 2017). Thus, gut microbiota has a possibly indirect effect on the pathophysiology of OA through affecting the metabolism of FFAs, however, the specific pathway in where needs to be further explored.

A variety of dietary factors have been reported to be involved in the pathophysiology of OA, such as saturated fatty acids, polyunsaturated fatty acids (PUFAs), antioxidants and amino acids (Li et al., 2016c; Sekar et al., 2017; Thomas et al., 2018). Saturated fatty acids have been demonstrated to have toxic effects on the etiology of OA (Sekar et al., 2017) and Baker et al. (2012) suggested that the systemic levels of n-3 and n-6 PUFAs controlled by diet might be related to articular cartilage composition and structural damage. Dietary antioxidants, such as vitamin C, D, and K, have been shown to possibly prevent the progression of OA through regulating collagen formation, bone metabolism, and cartilage mineralization (Thomas et al., 2018). Some gut microbial metabolites of aromatic amino acids (tyrosine, tryptophan and phenylalanine) were also considered to interact with host signaling pathways and thus affect host immunity (Ramadoss et al., 2005; Venkatesh et al., 2014; Boulange et al., 2016), which may accelerate the development of OA. Significantly, the structure and function of gut microbial community are closely related to diet (Wang et al., 2015; Velasquez, 2018). Schott et al. (2018) have showed that prebiotic fiber supplementation can reverse the harmful effects of fat-enriched (especially saturated fatty acids) diets on gut microbiota by increasing the abundance of beneficial bifidobacteria at the expense of multitudinous pro-inflammatory microorganisms. Kaliannan et al. (2015) found that high n-3 and high n-6 PUFAs diets each other antagonistically affected the proportion of pro-inflammatory bacteria and anti-inflammatory bacteria in mice models, which also suggested that the tissue n-3/n-6 PUFAs ratio altered gut microbiota profile composition and intestinal permeability. Moreover, some studies reported that high and low vitamin D diets affected the proportion of pro-inflammatory bacteria, particularly Bacteroidetes (Ooi et al., 2013; Assa et al., 2014; Ghaly et al., 2018), which showed that vitamin D modified the diversity of gut microbiota. Furthermore, dietary glutamine supplementation could modulate the composition and metabolism of gut microbiota, resulting in the change of Firmicutes/Bacteroidetes ratio (Ren et al., 2014b). Indole-3-propionate, a tryptophan-derived gut microbial metabolite (Russell et al., 2013), has also been found to improve intestinal barrier permeability through upregulating junctional proteins expression and downregulating TNF-α production (Venkatesh et al., 2014), which indirectly limited the translocation of antigens and pathogens and maintained the stability of gut microbiota. In addition, choline and carnitine, essential nutrients contained in many foods, could be metabolized into trimethylamine-N-oxide (TMAO) by the trimethylamine lyase system (CutC/D) and the carnitine Rieske-type oxygenase/reductase system (CntA/B and YeaW/X) in gut microbiota (Koeth et al., 2019) and subsequent flavin-containing monooxygenases in the liver (Canyelles et al., 2018). TMAO has been shown to be likely to increase TNF-α and IL-1β levels and decrease anti-inflammatory factor IL-10 levels (Ufnal et al., 2014), and be able to significantly trigger oxidative stress (Sun et al., 2016) that plays a pivotal role in the pathogenesis of OA (Paździor et al., 2019), through which TMAO may be involved in the pathological changes of OA. Notably, chondroitin sulfate, a symptomatic slow-acting drug widely used in OA, may have prebiotic properties (Rani et al., 2019) and can modulate the composition of gut microbiota such as Bacteroides (Shmagel et al., 2019). The composition of gut microbiota affected the effect of chondroitin sulfate on OA by influencing pro-inflammatory and anti-inflammatory activity of chondroitin sulfate on exacerbation or amelioration of OA *via* demolishment or reinforcement of the colonic mucus barrier, actions in where depended on the presence of specific symbiotic probiotics such as *A.muciniphila* (Wang et al., 2017).

### Bone Metabolism

**Figure 7:**
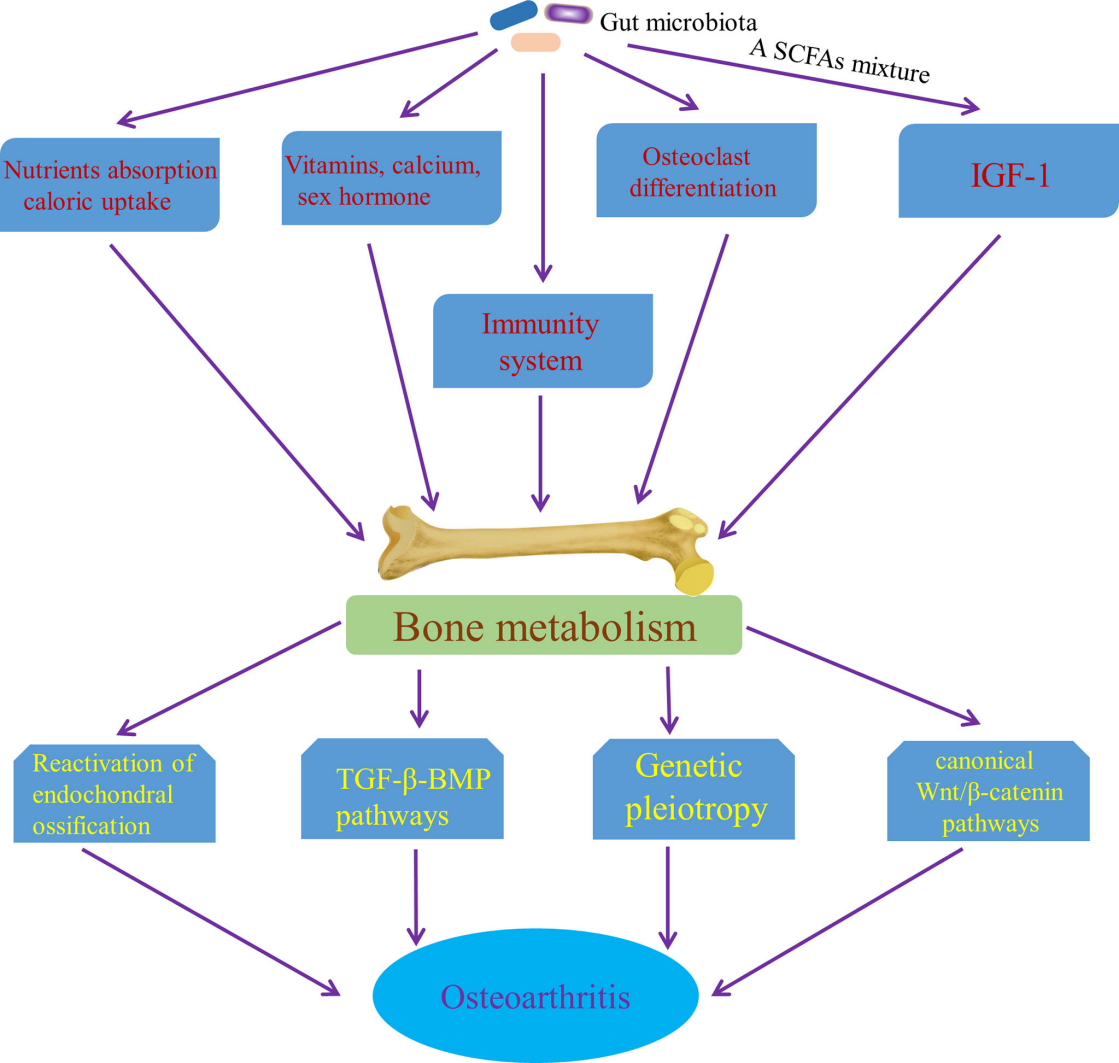
Pathways of the effect of gut microbiota on OA though affecting bone metabolism. SCFAs mixture, a short chain fatty acids mixture, containing acetate, butyrate and propionate; IGF-1, insulin growth factor-1; TGF-β-BMP pathway, the transforming growth factor β–bone morphogenetic protein signaling pathways.

Interestingly, the relationship between high bone mineral density (BMD) and OA have been consistently demonstrated by cross sectional and longitudinal epidemiological studies, which suggests that increased BMD is a risk factor for OA (Hardcastle et al., 2015). Although the results of some studies are controversial and contradictory, it may be due to confounding factors, such as differences in bone size (Javaid and Arden, 2013), which may confuse the relationship between BMD and OA. Some potential mechanisms underlying the relationship between BMD and OA have been proposed mechanisms. It has been speculated that high bone mass individuals show a trend of “bone-forming”, which increases their risk of OA (Hardcastle et al., 2014; Hardcastle et al., 2015). Moreover, in some studies that focus on the role of subchondral bone in cartilage loss, a characteristic of most OA, attenuation of the articular cartilage from below, due to reactivation of endochondral ossification at the bone–cartilage interface in OA joints that leads to tidemark duplication and advancement, has been report (Lories and Luyten, 2011; Burr and Gallant, 2012), which may an important mechanism of increasing bone formation driving OA procession. Also, soluble mediators released from bone may play a role in the articular cartilage through potential signaling pathways that includes the canonical Wnt/β-catenin signaling pathway and the transforming growth factor β (TGF­β)–bone morphogenetic protein (BMP) signaling pathways (Lories and Luyten, 2011), which provides another mechanism of bone formation affecting OA progression. Furthermore, genetic pleiotropy has been found to be possibly an important mechanism to explain the relationship between BMD and OA (Spector and MacGregor, 2004; Estrada et al., 2012; Yerges-Armstrong et al., 2014). Variation in BMD has been shown to be related to some OA susceptibility genes, the functional annotations of which are involved in bone-centred pathways such as skeletal development and morphogenesis, as well as osteoblast development/differentiation (Reynard and Loughlin, 2013). Genetic pleiotropy, genetic variants associated with both BMD and OA, could alter the risk of OA *via* mediated pleiotropy (Davey Smith and Hemani, 2014)—increased BMD itself or biological pleiotropy (Davey Smith and Hemani, 2014) that BMD genes increase the risk of developing OA by influencing other phenotypes, such as joint shape or cartilage thickness (Baker-LePain and Lane, 2010). Significantly, gut microbiota has an effect on the skeletal tissue though multiple mechanisms. Guss et al. (2017) concluded that alterations in the gut microbiota throughout growth impaired bone strength and influenced bone tissue mechanical properties. Also, gut microbiota has direct or indirect effects on bone metabolism by influencing nutrient absorption and caloric uptake (Hernandez et al., 2016) and changes in gut microbiota have been shown to contribute independently to bone growth and development (Blanton et al., 2016; Schwarzer et al., 2016). Gut microbiota affects the bone mass by effects on the metabolism of vitamins (Yatsunenko et al., 2012) and the status of sex hormone (Li et al., 2016a), as well as absorption of calcium (Yan et al., 2016). Sjögren et al. (2012) demonstrated that osteoclast differentiation was controlled by gut microbiota during skeletal growth. In the long term, a SCFAs mixture, containing acetate, butyrate and propionate, has also been found to increase the levels of insulin growth factor-1 (IGF-1) in serum and bone marrow, which coincides with anabolic effect on bone tissue (Yan et al., 2016). The evidence above suggested the anabolic effect of gut microbiota on bone tissue. Furthermore, in abnormal conditions, the immune system is the core of controlling BMD (Ibanez et al., 2019). For example, receptor activator of NF-κB ligand (RANKL) that is produced by mesenchymal cells, osteoblasts, and osteocytes in the bone marrow, as well as activated CD4+ T cells is the main cytokines involved in osteoclast differentiation (Ibanez et al., 2019). IL-17 and TNF-α can also stimulate osteoclastogenesis (Kotake et al., 1999; Lam et al., 2000). Ohlsson et al. (2017) has demonstrated that gut microbiota has a significantly effect on bone mass through NOD1 and NOD2 signaling pathway. MAMPs, distributed to bone organs, have been known to have a direct effect on bone remodeling through stimulation of innate immune receptors on bone cells, including TLR2 (Takami et al., 2002), TLR4 (Itoh et al., 2003) and TLR5 (Kassem et al., 2015). Collectively, it is possible that gut microbiota affects the occurrence and progress of OA by influencing bone tissue, especially anabolism of bone (**Figure 7**). However, this potential pathway is waiting for us to confirm.

## Microbiome-Gut-Brain Axis Is a Potential Pathway Involved in the Development of OA

**Figure 8:**
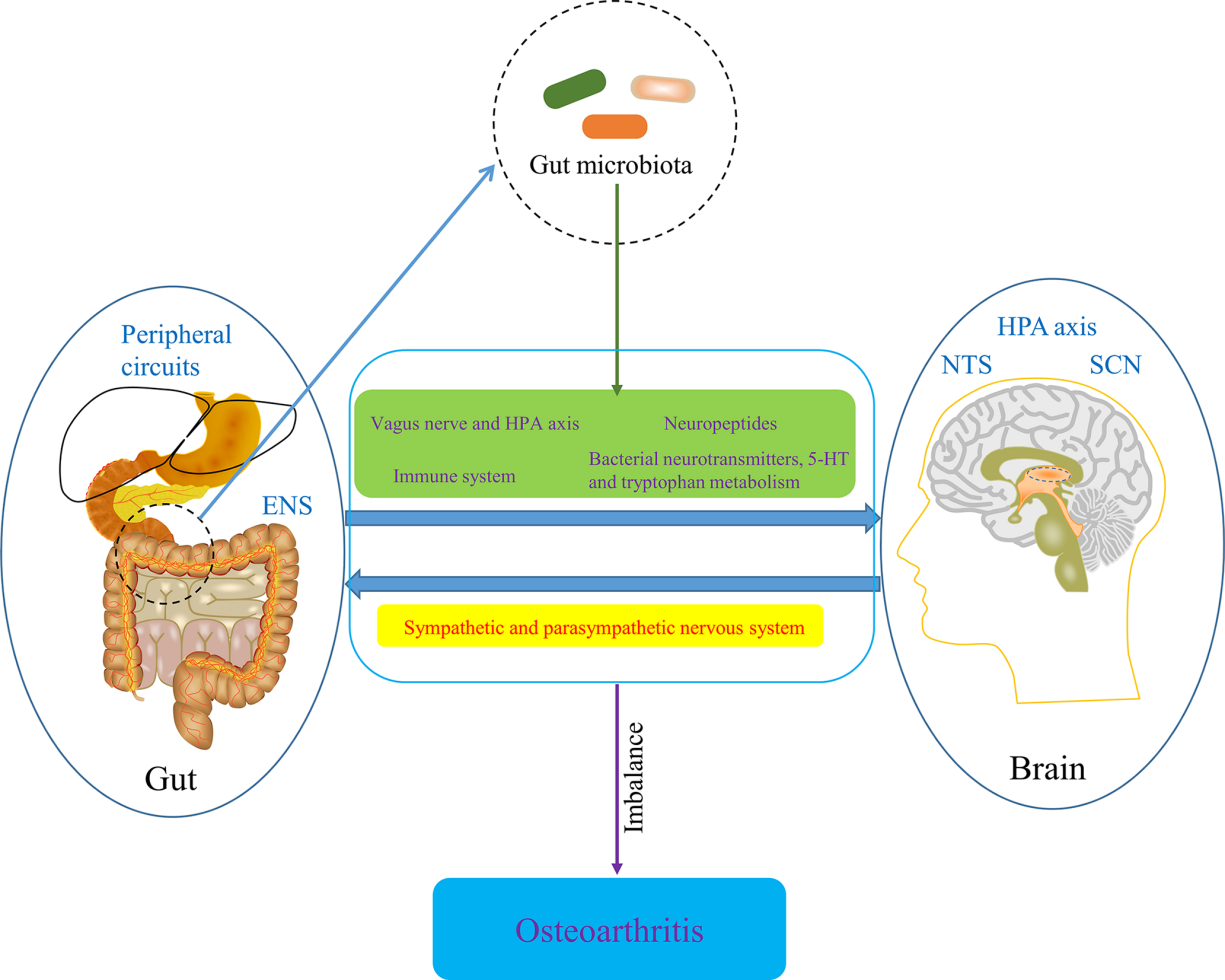
The potential role of microbiome-gut-brain axis in OA. ENS, enteric nervous system; HPA axis, hypothalamic-pituitary axis; NTS, nucleus tractus solitaries; SCN, hypothalamic suprachiasmatic nucleus.

The gut-brain axis is a bidirectional information communication system that integrates brain and gut functions. The bidirectional interaction between the Central nervous system (CNS), enteric nervous system and gastrointestinal tract, which embodies the gut-brain axis, is involved in the initiation and progression of numerous diseases. Some recent evidences have suggested that the gut-brain axis is involved in the development of OA. CNS has been known to play a role in OA pain and implications for rehabilitation (Murphy et al., 2012). With the development of neurophysiology, it has been known that the imbalance of body’s CNS and peripheral circuits is involved in OA progression (Dobson et al., 2018). Moreover, the CNS theory in OA pathophysiology is gradually refined, the new components of which includes hypothalamic-pituitary axis, nucleus tractus solitarius, hypothalamic suprachiasmatic nucleus and other associated higher centers. Each center has feedback circuits from intestinal tract (gut microbiota), OA joints and cellular metabolism. Circadian rhythms, gut microbiota, metabolism and redox regulation are controlled by central feed circuits above, the dysregulation of which is involved in the progression of OA (Morris et al., 2019). In addition, some information transmitted from intestinal tract is able to be distributed to the hypothalamus, regulating appetite, food intake, and energy expenditure (Silvestre et al., 2020), which is then involved in the progression of OA through the mechanism of metabolism mentioned in the previous part. Notably, the bidirectional communication between the brain and gut is significantly influenced by the gut microbiota (Caputi and Giron, 2018), so the concept of microbiome-gut-brain axis come in being. Collectively, CNS regulates gastrointestinal tract and ENS through sympathetic and parasympathetic nervous system, while gut microbiota affects CNS function through neural pathway, particularly vagus nerve and HPA axis, enteroendocrine signaling pathway, such as several neuropeptides that are produced by enteroendocrine cells stimulated by gut microbiota, and immune system, as well as bacterial neurotransmitters, especially serotonin [5-hydroxytryptamine (5-HT)] and tryptophan metabolism (Tillisch, 2014; Mayer et al., 2015; Foster et al., 2017). Also, SCFAs may contribute the regulation of OA-related pain by being involved in the gut-brain axis (Russo et al., 2018). Pain sensitization is the characteristic of chronic pain in osteoarthritis and hyperactivity of microglia in the spinal dorsal horn underlies the mechanisms of pain sensitization at central level in OA (Pan et al., 2021). Also, Appleton’s review suggested that microgliosis (activation of microglia) might be a treatment target for pain sensitization in OA (Appleton, 2017). Notably, acetate, an essential gut microbiota-derived SCFAs, drives microglia maturation and regulates the homeostatic metabolic state during health and perturbation (Erny et al., 2021), which may be an important involvement of microbiome-gut-brain axis in OA development. Furthermore, given that Liang et al. found the circadian rhythmicity of some fecal bacteria, gut microbiota may interact with the circadian clock of host to regulate cartilage homeostasis (Leone et al., 2015; Liang et al., 2015). Taken together, microbiome-gut-brain axis is a potential pathway involved in OA and restoring the balance of gut-brain axis by targeting gut microbiota may contribute to the amelioration of conditions of OA (**Figure 8**).

## Gut Microbiota Modulation: A New Therapy for OA

OA is a chronic debilitating disease, seriously affecting the quality of life of patients. However, in view of the slow progression of disease-modifying OA drugs (DMOADs), at present, the only accepted and available clinical method of treatment for OA is palliative care—that is, symptom palliation is the only alternative (Ghouri and Conaghan, 2019). Notably, although many factors are involved in the development of OA, gut microbiota has been regarded as an important pathogenic factor the initiation and progression of OA and the mechanisms underlying that gut microbiota is involved in OA are gradually elucidated. In addition, seeing that gut-muscle axis has an important role in the management of sarcopenia in inflammatory bowel disease, Nardone et al. (2021) once hypothesized that gut microbiota targeted treatment or complementary therapy could be implemented in patients with inflammatory bowel disease and sarcopenia. Therefore, gut microbiota modulation can also be proposed as a new therapy for OA. The good news is that several factors have been reported to be able to modulate gut microbiota and then modify OA.

### Probiotics and Prebiotics

Probiotics and prebiotics are the safe and effective dietary substances available, which can modulation the gut microbiota of the host by directly or indirectly promoting the growth of beneficial bacteria (Dahiya et al., 2017). Henrotin et al. (2019) showed that oral administration of Bifidobacterium longum CBi0703 over a period of 12 weeks decreased cartilage structure lesions and decreased type II collagen degradation, which suggested a potential prophylactic effect on OA development. Jhun et al. (2021) also suggested that oral administration of Lactobacillus rhamnosus ameliorated the progression of OA by suppressing joint pain and inflammation. Lei et al. (2017) indicated that Lactobacillus casei Shirota had beneficial effects on the treatment for knee OA in a randomized double-blind clinical trial. So et al. (2011) demonstrated that Lactobacillus casei could act as a powerful nutraceutical modulator for OA treatment by reducing pain, inflammatory responses and articular cartilage degradation. They also found that Lactobacillus casei with type II collagen/glucosamine synergistically significantly inhibited inflammation in OA through decreasing the expression of pro-inflammatory cytokines and MMPs, and upregulating anti-inflammatory cytokines. (Korotkyi et al., 2019) reported that probiotics upregulated the expression of type II collagen fiber alpha 1, a major gene encoding articular cartilage proteins that underlie cartilage tissue repair. Arora et al. (2021) indicated that probiotics can be available for mitigating symptoms (pain) and/or delaying pathological progression (joint degeneration) of OA. In the review article of Arora et al., it also was profiled that probiotics played a significant role in the key effect signaling pathways of OA *via* inhibition of vascular endothelial growth factor signaling, reduction of serum C-reactive protein (CRP) levels, reduction of oxidative stress and regulation of inflammatory markers in OA, such as pro-inflammatory cytokines and MMPs (Arora et al., 2021). In addition, some evidences from animal studies showed that prebiotics ameliorated OA status through modifying gut microbiota. Rios et al. (2019) showed that prebiotic oligofructose reversed the adverse effects of the intake of a high-fat/high-sucrose diet on knee joint of rats and significantly reduced knee joint damage, microbial dysbiosis, endotoxin levels and insulin resistance. Schott et al. (2018) also indicated that prebiotic oligofructose reversed the deleterious effects of high-fat diet-induced obesity on the gut microbiota and increased the abundance of beneficial Bifidobacteria at the expense of numerous microbes implicated as pro-inflammatory, meanwhile inducing the reduction of metabolic inflammation and protecting the joints of mice with OA from articular cartilage degeneration. Collectively, probiotics and prebiotics therapeutics are an effective method of improving the life quality of patients with OA.

### Diet and Nutraceuticals

Diet has been known to act as an important factor of affecting gut microbiota. Nutrient in diet could modulate gut microbiota by altering the microenvironment of gut microbiota, such as composition and metabolism of gut microbiota, and immune responses of host (Li et al., 2016b). For example, L-arginine (Ren et al., 2014a) or L-glutamine (Ren et al., 2014b) has been shown to have significant influence on gut microbiota, such as the ratio of Firmicutes/Bacteroidetes. Glutamine promoted mouse intestinal secretory IgA (SIgA) production and IgA+ plasma cell numbers through immune pathways, which depended on the effect of glutamine on gut microbiota (Wu et al., 2016). Chitosan has been reported to decreases mice weight through its effect on gut microbiota (Xiao et al., 2016). Oral supplementation of resveratrol has also shown a significantly protective effects on joints in high-fat diet-induced OA mouse models through recovery of joint structure and type II collagen expression in cartilage, and inhibition of chondrocyte apoptosis (Gu et al., 2016), which is possibly involved in changes in gut microbiota. Moreover, Green-lipped mussel extract (Perna canaliculus) and glucosamine sulphate have both been demonstrated to improve symptoms of OA through the effect on the composition of gut microbiota (Coulson et al., 2013). Chondroitin sulfate disaccharides, a nutraceutical widely used to improve the symptoms of OA, have been shown to exert an anti-inflammatory effect by modifying the structure and function of the gut microbiota in mice under healthy and stressed conditions (Liu et al., 2017). Furthermore, pycnogenol, a proprietary extract from pine bark, was reported to mitigate the symptoms of OA through anti-inflammatory, antioxidant and cartilage protection effect after being metabolized by gut microbiota (Rohdewald, 2018). Taken together, we need to continue to explore the dietary factors and nutraceuticals that can affect the gut microbiota, and find out a new and nice treatment for OA.

### Exercise

Exercise is universally known to be beneficial to health. Exercise enhances butyrate producing bacteria, which reduce inflammation and promote cell proliferation (Matsumoto et al., 2008). Exercise can protect intestinal morphology and integrity, and reduce systemic inflammation, despite the presence of a high-fat diet, which suggests that exercise shows a unique gut microbiota independent of diet (Campbell et al., 2016). Some studies have shown that exercise can regulate the composition of gut microbiota (Monda et al., 2017) by reducing intestinal transit time (Cerda et al., 2016), inducing elevated intestinal IgA levels (Viloria et al., 2011; Macpherson et al., 2015), changing the distribution of bile acids (Cerda et al., 2016) and so on, enhance intestinal mucosal immunity (Cerda et al., 2016), increase the proportion of Bacteroides/Firmicutes (Evans et al., 2014), and increase the production of SCFAs (Evans et al., 2014; Hsu et al., 2015), which is potential mechanism of the effect of exercise on health. In addition, several specific microbiota taxa, such as Lactobacillus and Bifidobacterium, which can be positively modulated by exercise (Yin and Niu, 2010; Petriz et al., 2014), have potential value in the treatment for OA, as mentioned above. As a result, exercise can increase the number of beneficial microbial species and enrich the diversity of gut microbial community, which contribute to our health. Furthermore, after de Sire et al. (2020) reviewed some evidence supporting the hypothesis of gut–joint axis, they indicated physical exercise combined with nutraceuticals interventions might have a key role in the regulation of gut microbiota and could be considered as adjuvant treatments in OA. Notably, de Sire et al. (2021) suggested the synergistic effects of physical exercise and nutraceuticals interventions to counter OA by summarizing the involvement of physical exercise and nutraceuticals in molecular pathways of OA for apoptotic, pro-, or anti-inflammatory signaling. However, we need to further study the mechanism of exercise-induced and combination of exercise and other interventions-induced changes in the composition and function of gut microbiota and all related effects, especially the effect on OA.

### Fecal Microbiota Transplantation

Fecal microbiota transplantation (FMT) is an operation designed to treat diseases associated with the gut microbiota by transferring feces from a healthy donor to the distal gastrointestinal tract of a recipient (Cammarota et al., 2017). It is promising that FMT has been performed and successfully cured Clostridium difficile infection (Hamilton et al., 2012). Studies have also confirmed that FMT has a role in the treatment for gastrointestinal diseases, liver diseases, infectious diseases, nervous system diseases as well as cancers (Chen et al., 2019; Vaughn et al., 2019; Vendrik et al., 2020; Chauhan et al., 2021; Revolinski and Munoz-Price, 2021). Significantly, the study of Huang et al. (2020) showed that gut microbiota from different groups of people could change the pathological process of joint injury-induced OA in germ-free mice, which offers a bright future for the application of FMT in the treatment for OA. Given the direct effect of FMT on gut microbiota and the excellent promise of FMT, the treatment for OA by FMT is possible. However, there is few studies related to this field, so we should carry out studies widely to verify the feasibility of the treatment for OA with FMT. Meanwhile, we need to deal with such challenges as costs of donor identification and screening, standardization of material preparation, indications of FMT and host immune rejection.

## Limitations

Up to now, despite these evidences above, a causal link between gut microbiota and OA has not been established and the specific role of gut microbiota in OA is still far from being understood in detail. There are few direct clinical data on the role of gut microbiota in OA and most of the evidence linking gut microbiota and OA is mainly obtained from animal model experiments. In addition, there are mainly medium and high-quality evidences provided by the studies cited in our review.

## Conclusion and Future Perspectives

**Figure 9:**
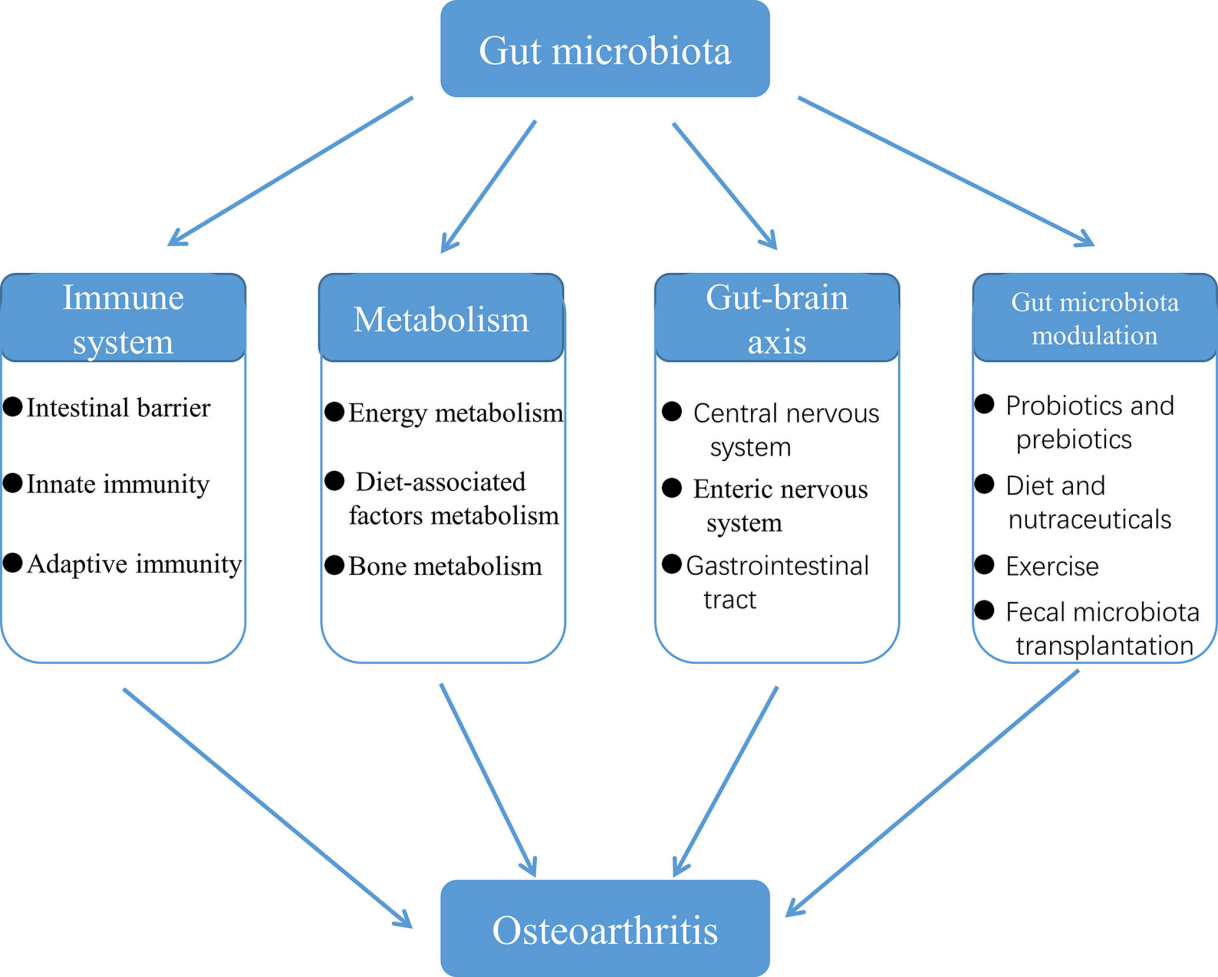
The role of the gut microbiota in the initiation and progression of OA.

OA that is difficult to cure is a global public health problem, the incidence and disability rate of which are both high. As a result, clarifying the potential mechanisms of OA pathogenesis has significant implications for our development of novel means of disease prevention and treatment. In this review, we have summarized the mechanisms of involvement of the gut microbiota in the initiation and progression of OA, and potential of gut microbiota modulation in the treatment for OA (**Figure 9**). However, due to the lack of a systematic search, screening and selection process of the corresponding studies, a few mechanisms of the involvement of gut microbiota in OA may be ignored by us. Furthermore, in order to provide a solid theoretical basis for the treatment for OA by gut microbiota, the potential mechanisms of the association between gut microbiota and OA need to be further explored and more studies are needed to understand the potential shared pathways and synergism between probiotics, diet, nutraceuticals and exercise in the regulation of the gut microbiota. In recent years, with the vigorous development of next-generation sequencing, transcriptomics and metabolomics, it has become more efficient and reliable to explore the changes in gut microbiota composition, bacterial diversity, and bacterial genes and metabolic pathways in patients with OA. It is promising to discover biomarkers associated with dysbiosis of OA, and specific pathogens and corresponding signal transduction pathways involved in the pathogenesis of OA, which make it possible to treat OA by targeting gut microbiota.

## Author Contributions

ZW wrote the article. FL and GP designed and reviewed the paper. All authors contributed to the article and approved the submitted version.

## Funding

This work was supported by the National Natural Science Foundation of China under Grant 81802164, Henan Key R&D Promotion Project under Grant 22170108, Medical Scientific and Technological Research Project of Henan Province under Grant SBGJ2018029, Youth Innovation Fund Project of the First Affiliated Hospital of Zhengzhou University under Grant YNQN2017040.

## Conflict of Interest

The authors declare that the research was conducted in the absence of any commercial or financial relationships that could be construed as a potential conflict of interest.

## Publisher’s Note

All claims expressed in this article are solely those of the authors and do not necessarily represent those of their affiliated organizations, or those of the publisher, the editors and the reviewers. Any product that may be evaluated in this article, or claim that may be made by its manufacturer, is not guaranteed or endorsed by the publisher.
